# Effectiveness of bony batten grafting for correction of caudal septal deviations

**DOI:** 10.1016/j.bjorl.2025.101573

**Published:** 2025-04-03

**Authors:** Nátalie Emy Yvamoto, Marco Antonio dos Anjos Corvo, Rodolfo Alexander Scalia, Eduardo Landini Lutaif Dolci

**Affiliations:** Santa Casa de Misericórdia de São Paulo Hospital, Departamento de Otorrinolaringologia, São Paulo, SP, Brazil

**Keywords:** Nasal obstruction, Nasal surgical procedure, Nasal septum, Grafts, Septal nasal cartilage

## Abstract

**Objective:**

The aim of this study was to retrospectively evaluate the efficacy of using the Batten septal bone graft intraoperatively to correct anterior septal deviations that covers Zone 1 and Zone 2 as proposed by Cottle using the NOSE scale.

**Methods:**

To this end, 30 patients with anterior septal deviations who underwent Functional Rhinoseptoplasty surgery from February 2019 to May 2023 and used septal bone grafting to correct the deviation were collected in a clinical study at the Otorhinolaryngology Department of a tertiary hospital in São Paulo.

**Results:**

There was a reduction in the Nose Scale from 75.5% to 17.3% in patients who underwent septal deviation correction using this graft (*p* < 0.001).

**Conclusion:**

The septal bone batten trought the endonasal approach proved to be an excellent alternative for correcting anterior deviations in patients where the simple removal of osteo-cartilaginous structures is not enough to resolve them. This graft is easy to obtain, has the ideal properties for its purpose and presented no complications related to its use in the study.

**Level of evidence:**

Level 2.

## Highlights

• Use of septal bone graft intraoperatively to correct anterior septal deviations.
• Reduction in the Nose Scale in patients who utilized Bone Septal Batten.
• The septal bone graft usually discarded in surgery can be used in rhinoseptoplasty.
• Bone grafting has low resorption rates and complications related to its use.

## Introduction

**Fig. 1:**
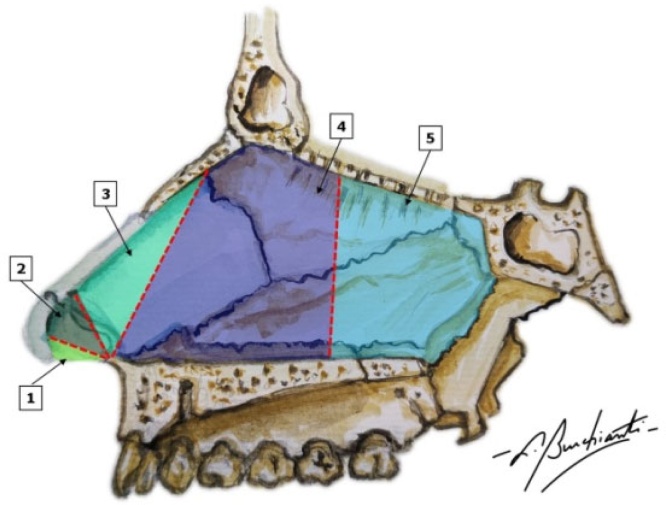
Schematic view of the lateral nasal septum according to the Cottle classification. (1) Area 1 ‒ Nasal or caudal vestibule; (2) Area 2 ‒ Internal nasal valve; (3) Area 3 ‒ Atrium; (4) Area 4 ‒ Turbinate’s region; (5) Area 5 ‒ Sphenopalatine region.

Septal deviations can be classified in different ways. One method focuses on the anatomical structure affected, categorizing them as cartilaginous and/or osseous, while another classification uses anatomical location, differentiating between anterior, posterior, inferior, superior and central.^1^ Of all the classifications, the one proposed by Cottle,^2^ who divided the septum into five regions, is the most widely used, as shown in the image (Fig. 1).

A study carried out on the cadavers of 2,000 patients showed that 75% had a deviated nasal septum.^3^ This shows the prevalence of this anatomical alteration, which can have a significant impact on quality of life, and can be congenital, traumatic or iatrogenic. The most frequent complaints in these patients are nasal obstruction and congestion, hyposmia, recurrent bouts of sinusitis, and tiredness when performing physical activities.

Septoplasty is the most common technique used to address a deviated septum, and was first described by Adams^4^ in 1875, who instituted the use of forceps to correct septal deviation. In 1936, Metzenbaum^5^ described an approach to caudal septal deviation involving relocation of a caudal strut on the maxillary crest.

Inferior and posterior deviations are the simplest deviations to correct, as they are usually resolved with simple osteo-cartilaginous removals. However, deviations located in the “L” strut, which are superior and/or anterior deviations, covering areas 1 and 2 according to the Cottle Classification, require different surgical techniques for their resolution, as simple removal is not applicable in these regions of septal support. In the upper region of the septum, spreader grafts are the standard treatment. For anterior deviations, several options are described in the literature, from more conservative techniques such as sutures and remodeling through incisions in the cartilage, and techniques that make use of grafts, to more invasive techniques such as extracorporeal septoplasty (first described by King and Ashley,^6^ and later by Gubish^7^), or modified anterior extracorporeal septoplasty described by Most.^8^ The simple removal of the caudal septum, carried out a few decades ago, is still performed by some surgeons today, and is a maneuver that should no longer be performed as it can lead to a loss of support for the nasal tip, resulting in ptosis, and frequently external nasal valve insufficiency, a condition in which airflow through the nose is compromised.

Described for the first time in 1966 by Dupont and colleagues,^9^ the septal batten is an excellent alternative for correcting anterior septal deviations, as it allows these obstructive anterior deviations to be resolved in a non-invasive and highly effective way.^10^, ^11^, ^12^, ^13^, ^14^ It can be made from septal cartilage, conchal cartilage, costal cartilage or bone, using a perpendicular blade from the ethmoid or vomer bone. With the advent of new technologies in nasal surgery, it is now possible to use a motor and drills to quickly and effectively shape a septal bone graft to be used as a septal batten. It needs to be rigid, not too thick and with perforations to nourish the septal cartilage and allow for suture placement.

Studies have shown that the use of a septal bone batten has low complication rates,^10^, ^14^, ^15^, ^16^ such as reduced air flow on the side where it is fixed,^10^, ^15^ as well as low resorption rates. A study by Kayabasoglu showed that the graft remained at 80%–90% of its initial size in 18 out of 21 patients in post-operative follow up using computerized tomography undertaken at least 12-months after the procedure.^16^

There are few studies in the literature describing the use of these grafts, although they are easy to make, most often removed and discarded during a septoplasty and have an excellent indication for the correction of complex anterior deviations.

The aim of this investigation was to retrospectively evaluate the efficacy of using septal bone grafts intraoperatively performed through a functional closed rhinoseptoplasty to correct anterior septal deviations in Zone 1 and/or Zone 2 as defined by Cottle^2^ using the Nasal Obstruction Symptom Evaluation (NOSE) scale. The scale contains five questions related to common nasal congestion problems which are scored on a scale of 0 (no problem) to 4 (severe problem); the final score is then multiplied by five to give a maximum score of 100.

## Methods

### Study design

This is a retrospective clinical study carried out in the otorhinolaryngology department of a tertiary hospital.

### Inclusion and exclusion criteria

#### Inclusion criteria

(1) Adults aged ≥17-years with nasal obstructive complaints who required functional rhinoseptoplasty surgery at the department of otorhinolaryngology, from February 2019 until May 2023, with septal bone batten placement to correct the deviation; (2) Patients who had an obstructive nasal septum deviation on preoperative physical examination that covered Zones 1 and/or 2 of the Cottle Classification^2^; (3) Patients who completed the NOSE Scale in the preoperative and postoperative periods (at least six months after the surgical procedure); (4) Patients submitted to functional closed rhinoseptoplasty through intercartilaginous and transfixing incisions; (5) Patients who agreed and retrospectively signed a free and informed consent form.

#### Exclusion criteria

(1) Patients who required other types of septal grafts to correct the deviation, such as spreader grafts; (2) Patients who did not attend post-operative follow-up for at least six months after the surgical procedure; (3) Patients submitted to open rhinoseptoplasty; (4) Patients who did not complete the NOSE scale pre-operatively; (5) Patients with any other otorhinolaryngological nasal disorders that, in the opinion of the evaluators, could interfere with the object of the study's evaluation, such as patients with nasosinusal diseases (polyps and polyposis) and synechiae; (6) Patients who did not have enough bone graft intraoperatively to make the study graft.

### Research procedures

A retrospective analysis was carried out of patients who underwent functional closed rhinoseptoplasty surgery from February 2019 until May 2023 at the Surgical Center of a tertiary hospital, and met the study eligibility criteria.

Data on the type of deviation prior to surgery, age when surgery was performed, gender, whether the deviation was caused by nasal trauma, previous nasal surgeries, simultaneous performance of turbinate surgery, pre- and post-surgery NOSE scores was collected from their medical records.

### Surgical technique

All patients were submitted to functional rhinoseptoplasty surgery by the hospital’s otorhinolaryngology team.

The surgical technique used had been individualized for each patient according to their complaints and a physical examination. All operations were performed using a closed access route (transfixing and intercartilaginous incision). The following procedures were performed: bilateral mucoperichondrial detachment; separation of the superior lateral cartilages from the quadrangular cartilage; removal of a portion of bone from the perpendicular lamina of the ethmoid, vomer or crest of the maxilla to make a bone batten; shaping of the graft with a micro motor (Beltec® Lb100) and drills (Razek® model BRD with a diameter of 3.1 mm to shape the bone graft, and model BP with a diameter of 1.0 mm to make perforations for passing wires); and fixed with at least two stitches of nylon 4.0 thread in the quadrangular cartilage of the septum to correct the caudal deviation.

### Financial investment

The surgeries and ambulatory care were carried out in a tertiary hospital belonging to the Public Health System (*Sistema Único de Saúde* – SUS). The drills and the motor used to make the septal bone batten grafts were privately owned and did not represent additional costs.

### Statistical analysis

For the sample size, it was used the student's *t*-test for paired samples, with a significance level of 5% and a test power of 80%, and used data from the article Surgical Outcomes of Bony Batten Grafting to Correct Caudal Septal Deviation in Septoplasty by Kim DY, Nam SH, Alharethy SE, Jang YJ.^11^ The software used to perform the statistical analysis was SPSS version 25.0 with a result of n = 4.

## Results

**Fig. 2:**
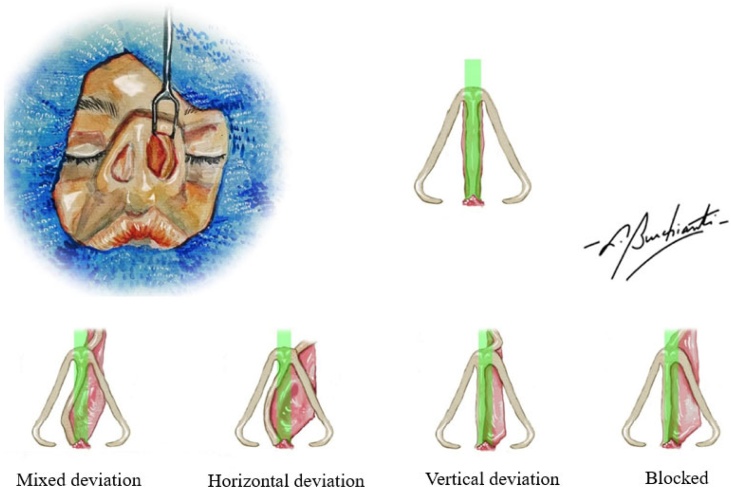
Anterior deviations found in patients. Mixed deviation: association of horizontal and vertical deviations. Horizontal deviation: deviation convexity with greater amplitude in the longitudinal axis. Vertical deviation: deviation convexity with greater amplitude in the vertical axis. Blocked: septum deviated from the central axis of the anterior nasal spine.

Our analysis identified 182 patients who attended Functional Rhinoplasty Ambulatory Clinic for pre- or post-operative consultations between January 2019 and March 2023 with anterior deviations in the pre-operative period (Fig. 2), and underwent surgery between February 2019 and May 2023.

**Fig. 3:**
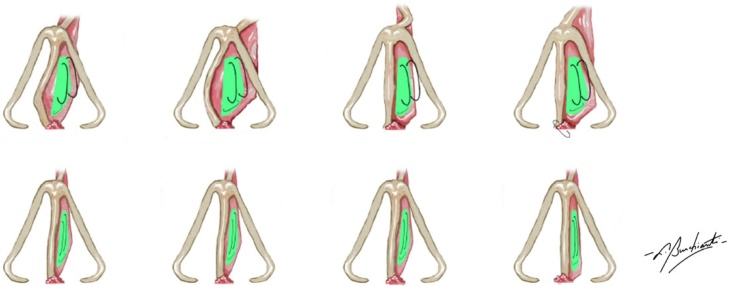
Fixing the bone batten to correct the deviations, preferably positioning the graft on the side contralateral to the deviation (concave side).

Of these 182 patients, 86 required the placement of a septal bone batten to correct the deviation (Fig. 3), but with 49 of these patients also requiring another type of graft to completely correct the deviation. Thus, 37 patients required only the placement of the bone batten during surgery. Of these, 7 did not complete the six months of post-operative follow-up and were excluded from this study, resulting in a total study sample of 30, who were, predominantly males (76.7%) with an average age of 30.5-years (17‒59-years). Eight of the 30 patients reported nasal trauma prior to surgery (26.7%) and 3 (10%) had previously undergone surgery to correct the deviation without the use of batten, resulting in unsatisfactory surgical outcomes and necessitating a nem approach.

**Fig. 4:**
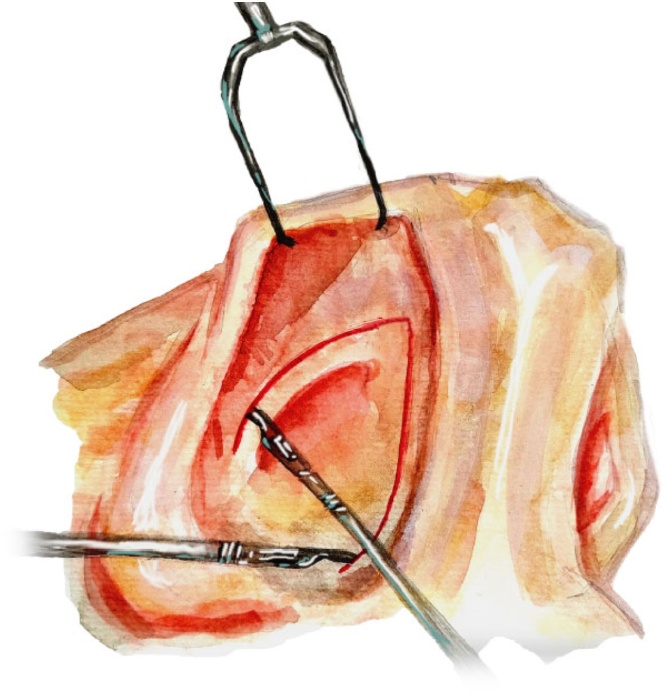
Closed access through intercartilaginous and transfixing incision.

Among the 30 patients (100%) who underwent surgery using a closed approach with an intercartilaginous and transfixing incision (Fig. 4), 13 (43.3%) also underwent turbinate surgery in the same surgical procedure of septal deviation correction.

**Fig. 5:**
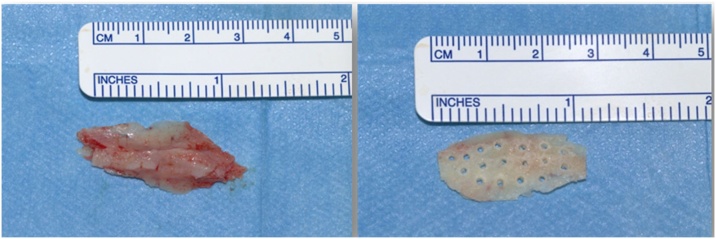
Left image: Bone removed for grafting. Image on the right: Bone prepared for use as a batten.

**Fig. 6:**
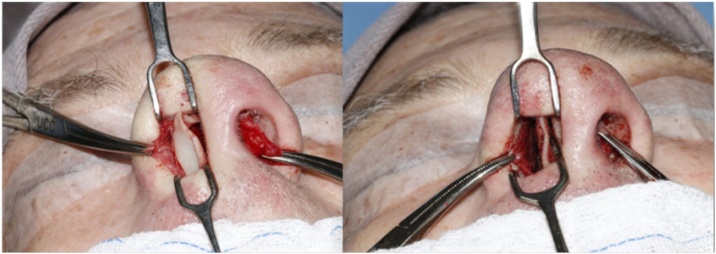
Patient with mixed deviation requiring a transverse position graft fixed to the quadrangular cartilage of the septum to correct the deviation.

In all the patients participating in the study, bone grafts were taken to make the bone Batten to correct the deviation (Fig. 5, Fig. 6).

With regard to the types of deviations found, we divided them into five types: (i) Zone 1 deviation to one side; (ii) Zone 1 deviation to one side with contralateral zone 2 vertical deviation; (iii) Zone 1 deviation to one side with contralateral zone 2 deviation in block up to zone IV; (iv) Zone 2 deviation (superior affecting internal nasal valve or isolated vertical deviation or isolated horizontal deviation); and (v) Deviation from zone 2 in block up to zone 4 on one side only.

**Fig. 7:**
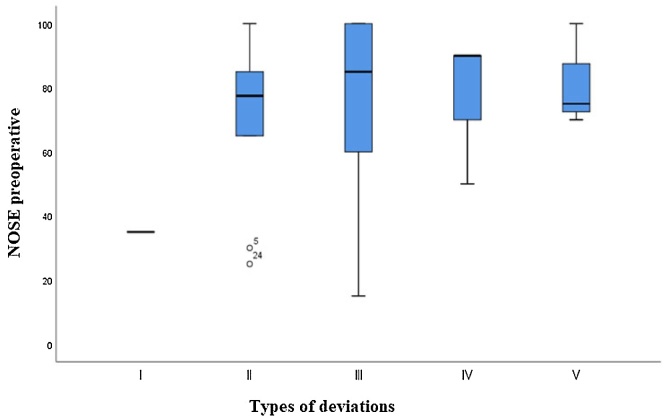
Boxplot of the preoperative NOSE score in relation to the type of deviation presented.

Of the 30 patients included in this study, one had a type I deviation, ten had a type II deviation, ten a type III deviation, six a type IV deviation, and three a Type V deviation. Analyzing these patients in terms of the results of the preoperative NOSE scale results: Type I had an average NOSE score of 35; Type II of 71.5; Type III of 79; Type IV of 80 and Type V of 81.7 (*p* = 0.857), and did not represent any statistically significant difference in terms of the preoperative NOSE score in relation to the type of deviation presented by the patient (Fig. 7).

**Fig. 8:**
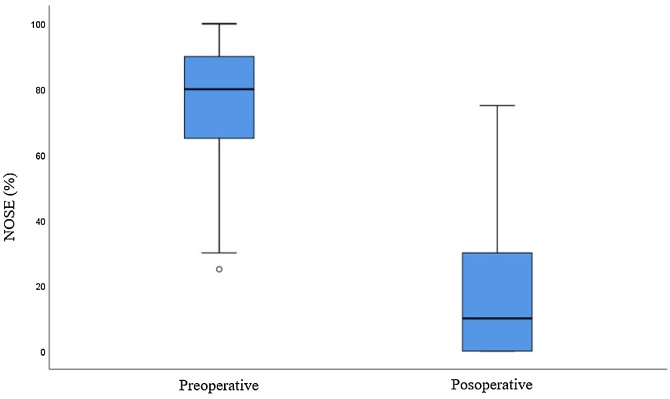
Boxplot of preoperative and postoperative nose.

The average preoperative NOSE score was 75.5 and the average postoperative score at least six months after surgery was 17.3 (*p* < 0.001). All patients analyzed in this study showed an improvement in the NOSE scale and reported an improvement in nasal obstruction when compared to the preoperative period (Fig. 8).

Of the 30 patients in the study, 13 were also submitted to turbinate reduction surgery associated with septal deviation correction. Although better NOSE results were observed in the patients who underwent turbinate surgery, the statistical difference was not significant (*p* = 0.180). The average of the NOSE score of patients who were not submitted to an associated turbinate reduction ranged from 60.14 to 13.61 in the post-operative period, and the average of those who were submitted to the combined procedure ranged from 62.3 to 13.54.

## Discussion

Anterior septal deviation, whether in zone I, zone II or in zones I and II, is still a challenge for many nose surgeons. Resolving this type of septal deviation is undoubtedly a necessity for every otorhinolaryngologist surgeon, as it is a deviation commonly found in the population.

The basic septoplasty technique, with a hemitransfixion incision, detachment of three Cottle tunnels and removal of the inferior portion of the quadrangular cartilage, maxillary crest and vomer is indicated in only 14% of patients who have only one inferior spur, according to the classification by Guyuron et al. based on the shape of the deviation, which has five other types, totaling six possible shapes.^17^

A systematic review of the options for treating caudal septal deviations by otorhinolaryngologists and facial plastic surgeons in North America reported that otorhinolaryngologists with a fellowship in facial plastic surgery perform these surgeries more often than general otorhinolaryngologists; and the most commonly used techniques are the swinging door (69.5%), extracorporeal septoplasty (46.7%), cartilage relaxing incisions (45.3%) and septal bone grafting (25.4%).^18^

Superior and anterior septal deviations require a more nuanced surgical approach than simply removing osteo-cartilaginous structures, as this does not resolve these deviations, and can cause the cartilaginous dorsum to collapse resulting in drooping of the nasal tip.

A systematic review showed that the main causes of failure in primary septoplasty are previously undiagnosed abnormalities of the nasal valve and insufficient correction of caudal septal deviations.^19^, ^20^

For the correction of these superior and/or anterior deviations, an excellent alternative, as proven in the current study, is the septal bone batten, which reduced the average NOSE score from 75.5 to 17.3 in patients who had their septal deviation corrected using this graft (*p* < 0.001). This method was first described in the literature in 1966 by Dupont et al.^9^ and, since then, other studies related to this technique have been published. The main advantages of this method is that the bone graft is easy to obtain and use, the material normally being discarded in surgeries after removal, and it has ideal properties for this purpose, such as rigidity and thickness.

The technique most commonly used consists of making a hemitransfixion or transfixion incision, creating a bone graft from the perpendicular lamina of the ethmoid or vomer bone, preparing the graft using drills or rasps, and fixing it in the region of the deviation using absorbable thread (PDS).^10^, ^11^, ^12^, ^13^, ^14^

Our study differs from previous studies in terms of the type of approach, as we opted to perform a closed rhinoseptoplasty with intercartilaginous and transfixing incisions. The purpose of performing this approach is to provide a wider exposure of the area affected by the deviation, and make it easier to separate the upper lateral cartilages of the nasal septum, facilitating graft fixation and also eliminating any possible extrinsic tension of the deviated septum.

We also opted to use a non-absorbable thread to fix the grafts, since the reabsorption of absorbable threads could cause alterations in the final result, such as recurrence of the deviation or mobilization of the septal batten graft. There were no cases of thread extrusion, or any type of infection related to the use of this type of suture, just as there were no complications related to the use of the described graft.

A possible limitation of the current study in respect of the NOSE scale results was the use of this graft with the simultaneous performance of turbinate surgery in some patients, since this procedure alone could have reduced the reported nasal obstruction. However, we found no statistically significant (*p* = 0.180) results in respect of the NOSE scores in the patients receiving one or both procedures, thus indicating that this did not compromise the results of the study. The same was observed in a study by Chung et al. in 2014,^10^ who reported a slight improvement in patients that also underwent turbinate reduction surgery in association with the correction of caudal septal deviation, but with no statistically significant difference.

## Conclusion

The septal bone batten proved to be an excellent alternative for correcting anterior septal deviations in patients where the simple removal of osteo-cartilaginous structures is not enough to resolve these deviations in the anterior region of the “L” strut. This graft has ideal properties in relation to its purpose, and there were no complications associated with its use.

## Declaration of competing interest

The authors declare no conflicts of interest.
